# Long-term outcomes of kidney transplant recipients with end-stage kidney disease attributed to presumed/advanced glomerulonephritis or unknown cause

**DOI:** 10.1038/s41598-018-27151-4

**Published:** 2018-06-13

**Authors:** Wai H. Lim, Germaine Wong, Stephen P. McDonald, Aron Chakera, Grant Luxton, Nicole M. Isbel, Helen L. Pilmore, Tom Barbour, Peter Hughes, Steven J. Chadban

**Affiliations:** 1 Department of Renal Medicine, Sir Charles Gairdner Hospital, Perth, Australia; 2 School of Medicine and Pharmacology, University of Western Australia, Perth, Australia; 3 Australia and New Zealand Dialysis and Transplant Registry, Adelaide, Australia; 4 Centre for Transplant and Renal Research, Westmead Hospital, Sydney, Australia; 5 Centre for Kidney Research, The Children’s Hospital at Westmead, Sydney, Australia; 6 Sydney School of Public Health, University of Sydney, Sydney, Australia; 7 South Australia Health and Medical Research Institute, Adelaide, Australia and University of Adelaide, Adelaide, Australia; 8 Department of Renal Medicine, Prince of Wales Hospital, Sydney, Australia; 9 Department of Nephrology, Princess Alexandra Hospital, Brisbane, Australia; 10 Renal Unit, Auckland Hospital & Department of Medicine, Auckland University, Auckland, New Zealand; 11 Department of Nephrology, Royal Melbourne Hospital and Department of Medicine, University of Melbourne, Melbourne, Australia; 12 Renal Medicine, Royal Prince Alfred Hospital, Sydney, Australia; 13 Kidney Node, Charles Perkins Centre, University of Sydney, Sydney, Australia

## Abstract

People with biopsy-proven glomerulonephritis (GN) as their cause of end-stage kidney disease (ESKD) who undergo kidney transplantation incur significant risk of recurrent GN-related graft failure, but the risk in recipients with ESKD where GN was suspected but not biopsy proven (presumed/advanced GN) and when the cause of ESKD is unknown remains uncertain. Using the Australia and New Zealand Dialysis and Transplant registry, we examined the associations between primary kidney transplant recipients whose ESKD was attributed to: 1) commonly-recurring GN (i.e. IgA nephropathy, membranoproliferative GN, focal segmental glomerulosclerosis and membranous GN), 2) presumed/advanced GN, and 3) cause of ESKD unknown (uESKD) and GN-related graft failure using adjusted competing risk models. Of 5258 recipients followed for a median of 8 years, 3539 (67.3%) had commonly-recurring GN, 1195 (22.7%) presumed/advanced GN, and 524 (10.0%) uESKD. Compared to recipients with commonly-recurring GN, recipients with presumed/advanced GN or uESKD experienced a low incidence of GN-related graft failure (<1%) and a lower hazard of GN-related graft failure (adjusted sub-distribution hazard ratio [HR] 0.28 [95%CI 0.15-0.54,p < 0.001] and 0.20 [95%CI 0.06-0.64,p = 0.007], respectively). People with ESKD attributed to either presumed/advanced GN or unknown cause face a very low risk of graft failure secondary to GN recurrence after transplantation.

## Introduction

Primary glomerulonephritis (GN) is one of the leading causes of end-stage kidney disease (ESKD) in Australia and worldwide, affecting up to 20% of patients commencing renal replacement therapy^1,2^ and up to 40% of those who receive a kidney transplant^3–6^. Disease recurrence after kidney transplantation is an important cause of graft loss^7,8^ and the ability to inform patients of this risk is one reason why clinicians seek to establish a histological diagnosis in those with ESKD that may be caused by GN^9^. In practice, a histological diagnosis is frequently not obtained. In some cases with clinical features of GN, a biopsy is not done due to patient refusal or the presence of contra-indications to biopsy such as clotting disorders or small kidneys, and such cases are frequently labelled “presumed GN”. In other cases the biopsy may not show clear features of any particular GN but only advanced interstitial scarring and glomerulosclerosis and in such cases ESRD is frequently attributed to “advanced GN”. In a subgroup of patients with ESKD, the underlying etiology of ESKD is unknown. The incidence of patients with ESKD attributed to presumed GN or “unknown etiology” varies widely, with reported incidence of up to 10%^10^.

Several types of GN including IgA nephropathy, membranoproliferative glomerulonephritis (MPGN), focal segmental glomerulosclerosis (FSGS) and idiopathic membranous nephropathy (IMN) are known to recur frequently post-transplantation (i.e. commonly-recurring GN), with up to 50% of those with recurrence experiencing graft failure after a median of 5 years^3,7,11–13^. The risks of GN recurrence and recurrence causing graft loss after transplantation remain unknown for patients with presumed or advanced GN and those with an unknown cause of ESKD. The risk of this outcome, placed in the context of other “competing” causes of graft failure including acute rejection, chronic allograft nephropathy and death with graft function, is important for patients and their physicians to understand. We sought to test the hypothesis that patients with ESKD secondary to presumed or advanced GN or an unknown cause have a low risk of GN-related graft failure compared to those with commonly-recurring GN. The primary aim of this study was to compare the risks of GN-related graft failure between kidney transplant recipients with presumed/advanced GN, ESKD from an unknown cause (uESKD) and those with ESKD secondary to biopsy-proven commonly-recurring GN, using data from the Australia and New Zealand Dialysis and Transplant (ANZDATA) registry.

## Results

### Study population

There were 5258 recipients included in this study, of whom 3539 (67.3%) had commonly-recurring GN, 1195 (22.7%) had presumed/advanced GN, and 524 (10.0%) had uESKD. Baseline characteristics of the study population according to exposure groups are shown in Table 1. The median (IQR) follow-up period for the cohort was 7.8(9.3) years resulting in 45,363 patient-years, with similar follow-up periods for recipients in all groups.

**Table 1:** Baseline characteristic of live- and deceased donor kidney transplant recipients stratified by categories of end-stage kidney disease between 1990–2012 (n = 5258).

	Commonly recurring GN (n = 3539)	Presumed/advanced GN (n = 1195)	uESKD (n = 524)	p-value
Demographics				
Age (years, mean±SD)	43.7±13.6	45.3±14.9	46.6±15.3	<0.001
Male (n, %)	2525 (71.3)	761 (63.7)	340 (64.9)	<0.001
Ethnicity (n, %)				<0.001
Caucasian	2845 (80.4)	868 (72.6)	358 (68.4)	
Indigenous	205 (5.8)	156 (13.1)	83 (15.8)	
Others	489 (13.8)	171 (14.3)	83 (15.8)	
Coronary artery disease (n, %)				<0.001
No	3248 (91.8)	1036 (86.7)	464 (88.5)	
Yes	242 (6.8)	102 (8.5)	41 (7.8)	
Missing data	49 (1.4)	57 (4.8)	19 (3.6)	
Peripheral vascular disease (n, %)				<0.001
No	3424 (96.8)	1109 (92.8)	494 (94.3)	
Yes	76 (2.1)	33 (2.8)	12 (2.3)	
Missing data	39 (1.1)	53 (4.4)	18 (3.4)	
Diabetes (n, %)	163 (4.6)	70 (5.9)	41 (7.8)	0.004
Body mass index (kg/m^2^, n, %)				<0.001
<20	363 (10.3)	154 (12.9)	57 (10.9)	
20.0–24.9	1321 (37.3)	393 (32.9)	186 (35.5)	
25.0–29.9	1081 (30.5)	298 (24.9)	145 (27.7)	
≥30	529 (14.9)	191 (16.0)	73 (13.9)	
Missing data	245 (7.0)	159 (13.3)	63 (12.0)	
Waiting time (years, mean±SD)	2.5±2.5	2.9±2.8	2.4±2.3	<0.001
Smoker (n, %)				<0.001
Non-smoker	1964 (55.6)	592 (49.5)	267 (51.0)	
Former smoker	1067 (30.1)	349 (29.2)	144 (27.5)	
Current smoker	347 (9.8)	145 (12.2)	69 (13.2)	
Missing data	161 (4.5)	109 (9.1)	44 (8.4)	
Biopsy of native kidneys (n, %)				<0.001
Yes	3539 (100.0)	231 (19.3)	66 (12.6)	
No/unknown	0 (0.0)	964 (80.7)	458 (87.4)	
GN types (n, %)				
IgA nephropathy	1980 (55.9)	0 (0.0)	0 (0.0)	
Membranous	264 (7.5)	0 (0.0)	0 (0.0)	
MPGN	257 (7.3)	0 (0.0)	0 (0.0)	
Primary FSGS	97 (2.7)	0 (0.0)	0 (0.0)	
Focal and segmental proliferative GN	200 (5.7)	0 (0.0)	0 (0.0)	
Focal sclerosing GN (±hyalinosis)	741 (20.9)	0 (0.0)	0 (0.0)	
Presumed GN	0 (0.0)	958 (80.2)	0 (0.0)	
Advanced GN	0 (0.0)	237 (19.8)	0 (0.0)	
Donor characteristics				
Age (years, mean±SD)	44.8±15.5	43.5±16.6	45.0±15.5	0.076
Type (n, %)				<0.001
Live-donor	1362 (38.5)	356 (30.0)	173 (33.1)	
Deceased donor	2173 (61.5)	832 (70.0)	349 (66.9)	
ABO-incompatible (n, %)	61 (1.7)	8 (0.7)	8 (1.5)	0.032
Immunology/Transplant				
HLA-ABDR mismatches (n, %)^*^				0.637
0–2	1372 (38.8)	456 (38.1)	208 (39.7)	
3–6	2154 (60.9)	732 (61.3)	312 (59.5)	
Missing data	13 (0.3)	7 (0.6)	4 (0.8)	
Peak PRA >50% (n, %)^#^	251 (7.1)	126 (10.5)	36 (6.9)	0.001
Total ischemic time (hours, mean±SD)^§^	9.7±7.2	11.4±7.4	11.1±7.3	<0.001
Induction (n, %)	1565 (44.2)	472 (39.5)	239 (45.6)	0.009
Transplant era (n, %)				<0.001
1990–1997	928 (26.2)	414 (34.6)	143 (27.3)	
1998–2005	1267 (35.8)	395 (33.1)	165 (31.5)	
2006–2012	1344 (38.0)	386 (32.3)	216 (41.2)	
Initial immunosuppression (n, %)				
Prednisolone	2979 (96.1)	910 (95.0)	410 (94.0)	0.065
CNI				0.376
None	104 (3.4)	36 (3.8)	11 (2.5)	
Cyclosporin	1907 (61.5)	564 (58.9)	258 (59.2)	
Tacrolimus	1088 (35.1)	358 (37.3)	167 (38.3)	
Anti-metabolite				0.024
None	217 (7.0)	60 (6.3)	25 (5.7)	
Azathioprine	484 (15.6)	192 (20.0)	73 (16.7)	
MMF/Myfortic	2398 (77.4)	706 (73.7)	338 (77.6)	

Data expressed as number (proportion) or as mean ± SD. GN - glomerulonephritis, HLA – human leukocyte antigen, PRA – panel reactive antibody, CNI – calcineurin-inhibitor, MMF – mycophenolate mofetil, uESKD – unknown etiology of end-stage kidney disease. Covariates with missing data: ^*^HLA-ABDR mismatches (n=18), ^#^peak PRA (n=22), and ^§^total ischemic time (n=118).

Mean recipient age and the proportion of deceased donor kidney transplants were greater in recipients with presumed/advanced GN or uESKD as compared to those with commonly-recurring GN. A greater proportion of recipients with presumed/advanced GN or uESKD were indigenous (13–16% vs. 6% with commonly-recurring GN). Biopsies were undertaken to establish ESKD in 19% and 13% of those with presumed/advanced GN and uESKD, respectively, compared to 100% of recipients with commonly-recurring GN. Variability in biopsy practices across Australia and New Zealand are likely, however the ANZDATA registry does not capture these data. The GN subtypes for recipients with commonly-recurring GN are shown in Table 1.

### Association between causes of ESKD and GN-related DCGF

**Figure 1:**
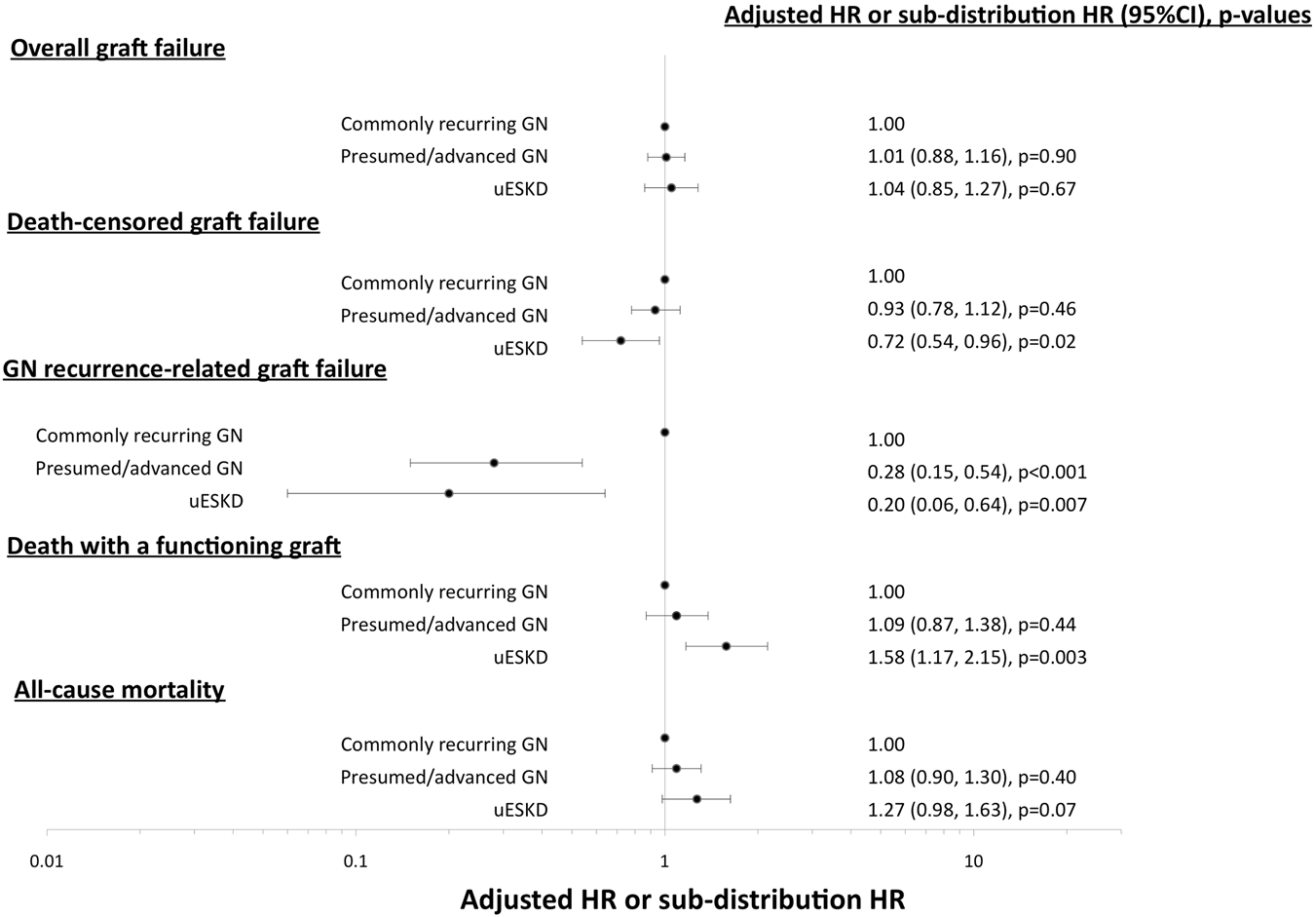
Forest plots showing the adjusted hazard ratios (HR) with 95% confidence intervals (95%CI) or subdistribution HR with 95%CI of the association between categories of end-stage kidney disease, overall graft failure, death censored graft failure, glomerulonephritis (GN)-related graft failure, death with a functioning graft and overall mortality. Cox regression and competing risk models were adjusted for donor and recipient age, ethnicity, era, waiting time and HLA-mismatches.

**Figure 2:**
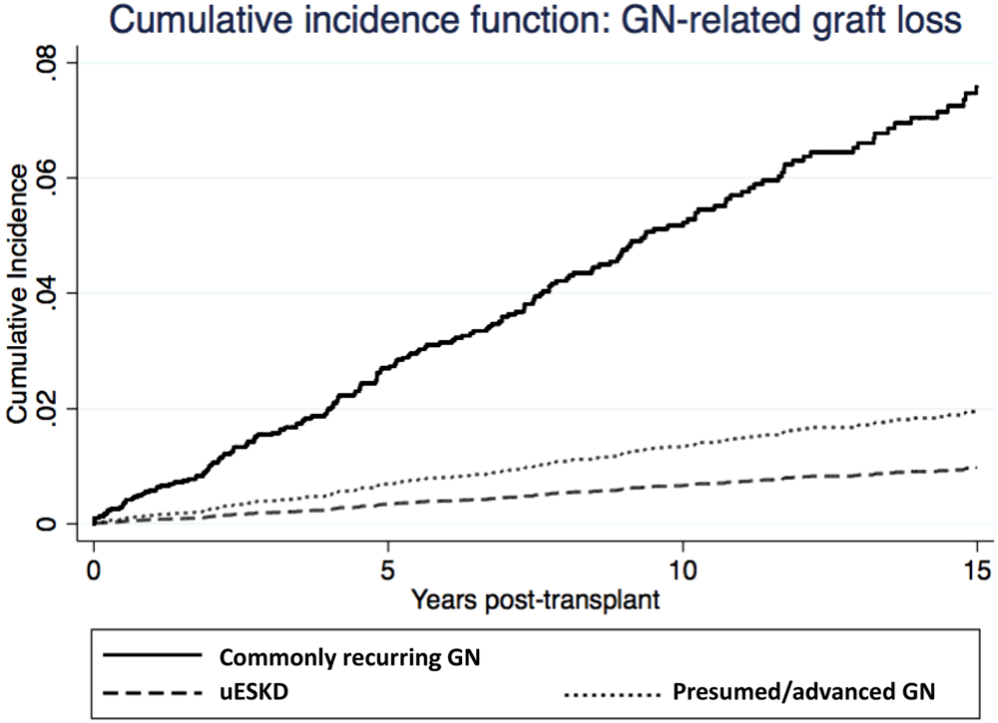
Cumulative incidence function curves of glomerulonephritis (GN)-related graft failure after kidney transplantation stratified by categories of end-stage kidney disease, adjusted for the competing risk of non-glomerulonephritis-related graft failure.

A greater proportion of recipients with commonly-recurring GN experienced graft failure from GN recurrence compared to recipients with presumed/advanced GN or uESKD (5%, 1% and 1%, respectively, p < 0.001). Compared to recipients with commonly-recurring GN, recipients with ESKD attributed to presumed/advanced GN and uESKD were less likely to experience GN-related DCGF with adjusted sub-distribution HR of 0.28 (0.15–0.54, p < 0.001) and 0.20 (0.06–0.64, p = 0.007), respectively (Table 2 and Fig. 1). Figure 2 shows the cumulative incidence of GN-related DCGF, stratified by ESKD groups, after adjusting for competing risks of non-GN-related causes of DCGF.

**Table 2:** Associations between categories of end-stage kidney disease, overall graft failure, death-censored graft failure and glomerulonephritis-related graft failure.

	Overall graft failure (HR, 95%CI)*	DCGF (sub-distribution HR, 95%CI)^#^	GN-related graft failure (sub-distribution HR, 95%CI) ^#^
ESKD types			
Commonly recurring GN	1.00	1.00	1.00
Presumed/advanced GN	1.03 (0.90, 1.18)	0.93 (0.78, 1.12)	0.28 (0.15, 0.54)
uESKD	1.04 (0.86, 1.26)	0.72 (0.54, 0.96)	0.20 (0.06, 0.64)
HLA-mismatch			
0–2 mismatches	1.00	1.00	1.00
4–6 mismatches	1.18 (1.04, 1.33)	1.27 (1.09, 1.49)	0.91 (0.63, 1.32)
Diabetes	—	0.98 (0.64, 1.51)	1.86 (0.71, 4.85)
Deceased donor (vs. live donor)	—	1.07 (0.79, 1.45)	0.87 (0.42, 1.80)
Ischemic time (per hour increase)	1.01 (1.00, 1.03)	1.01 (0.99, 1.03)	1.00 (0.50, 2.00)
Coronary artery disease	1.24 (1.00, 1.53)	1.16 (0.84, 1.61)	1.16 (0.49, 2.76)
Peripheral vascular disease	1.93 (1.42, 2.63)	1.80 (1.10, 2.95)	2.01 (0.62, 6.55)
Race			
Caucasian	1.00	1.00	1.00
Indigenous	1.96 (1.62, 2.37)	2.00 (1.57, 2.54)	1.33 (0.68, 2.58)
Others	1.03 (0.86, 1.24)	1.17 (0.93, 1.46)	0.89 (0.50, 1.59)
Smoking			
Non-smoker	1.00	1.00	1.00
Former smoker	1.29 (1.13, 1.47)	1.28 (1.08, 1.53)	2.00 (1.34, 2.98)
Current smoker	1.72 (1.44, 2.04)	1.61 (1.29, 2.02)	1.29 (0.70, 2.36)
Recipient age (per year increase)	—	0.97 (0.96, 0.97)	0.96 (0.95, 0.98)
Donor age (per year increase)	1.02 (1.01, 1.02)	1.02 (1.02, 1.03)	0.99 (0.98, 1.00)
Waiting time (per year increase)	1.06 (1.03, 1.08)	1.05 (1.02, 1.09)	1.04 (0.96, 1.13)
Peak PRA			
0–10%	1.00	1.00	1.00
11–50%	1.11 (0.95, 1.28)	1.02 (0.83, 1.24)	1.12 (0.70, 1.79)
>50%	1.40 (1.15, 1.72)	1.51 (1.16, 1.96)	1.28 (0.63, 2.61)
Transplant era			
1990–1997	1.00	1.00	1.00
1998–2005	0.62 (0.54, 0.72)	0.59 (0.50, 0.70)	0.52 (0.34, 0.78)
2006–2012	0.53 (0.42, 0.65)	0.50 (0.39, 0.64)	0.63 (0.37, 1.07)

Data presented as adjusted hazard ratio (HR) with 95% confidence interval (95%CI) from Cox regression models (*for overall graft failure) or as adjusted sub-distribution HR (95%CI) from competing risk models (^#^for death censored graft failure and GN-related graft failure). Only covariates remaining in the most parsimonious model are shown. GN – glomerulonephritis, HLA – human leukocyte antigen, ESKD – end-stage kidney disease, PRA – panel reactive antibody, BMI – body mass index, DCGF – death-censored graft failure, CNI – calcineurin-inhibitor, uESKD – unknown etiology of end-stage kidney disease, HR – hazard ratio.

For recipients with commonly-recurring GN, 164 (20.8%) cases of DCGF were attributed to recurrent GN (Table 3). Only 3/164 (1.8%) of GN-related DCGF in this group were attributed to non-commonly-recurring GN subtypes. For recipients with presumed/advanced GN or uESKD, 15 (5.4%) and 3 (3.3%) of DCGF were attributed to recurrent/de novo GN. The majority of GN-related DCGF in recipients with presumed/advanced GN (13/15 [86.7%]) or uESKD (2/3 [66.7%]) were attributed to commonly-recurring GN subtypes. Of the 46 (1.3%) patients with commonly-recurring GN who did not have kidney biopsies (and excluded from the study cohort), no patients developed GN-related graft failure.

**Table 3:** Causes of death-censored graft failure stratified by categories of end-stage kidney disease.

Causes of graft loss	Commonly recurring GN (n = 787)	Presumed/advanced GN (n = 278)	uESKD (n = 91)
CAN/IFTA (n, %)	402 (51.1)	142 (51.1)	47 (51.6)
Rejection (n, %)	47 (6.0)	29 (10.4)	9 (9.9)
Recurrence/de novo GN (n, %)	164 (20.8)	15 (5.4)	3 (3.3)
IgA nephropathy (n)	68	3	1
Membranous (n)	17	3	1
MPGN (n)	28	0	0
FSGS (n)	48	7	0
Other GN	3	2	1
HUS	4 (0.5)	3 (1.1)	0 (0.0)
Non-adherence (n, %)	26 (3.3)	23 (8.3)	9 (9.9)
Vascular complications (n, %)	56 (7.1)	23 (8.3)	5 (5.5)
Others (n, %)	88 (11.2)	43 (15.4)	18 (19.8)
BKVAN (n)	9	1	1
Haemorrhage (n)	6	2	2
Infection (n)	7	6	2
Ureteric/bladder complications (n)	3	2	2
Drug complications/withdrawal (n)	13	6	2
Non-functioning kidney (n)	5	3	2
Cortical necrosis post-transplant (n)	8	3	3
Miscellaneous (n)	37	20	4

Data presented as number (%) for the entire cohort (n=5258), p < 0.001 (across groups). Chronic allograft nephropathy/interstitial fibrosis and tubular atrophy (CAN/IFTA), GN – glomerulonephritis, MPGN – membranoproliferative glomerulonephritis, FSGS – focal segmental glomerulosclerosis, uESKD – unknown etiology of end-stage kidney disease, BKVAN – BK viral allograft nephropathy.

### Association between causes of ESKD and overall graft failure

Overall graft survival at 5 and 10 years for recipients with commonly-recurring GN was 84% (82–85%) and 67% (65–69%), respectively. This compared with 79% (76–81%) and 60% (57–64%), respectively for recipients with presumed/advanced GN; and 79% (75–83%) and 63% (57–68%), respectively for recipients with uESKD (log-rank p < 0.001). In the unadjusted model, recipients with presumed/advanced GN or uESKD experienced a higher risk of overall graft failure with unadjusted HR of 1.24 (1.12–1.38, p < 0.001) and 1.30 (1.12–1.51, p = 0.001), respectively compared to recipients with commonly-recurring GN. However, these associations were no longer significant in the adjusted model (Table 2 and Fig. 1).

### Association between causes of ESKD and DCGF

**Figure 3:**
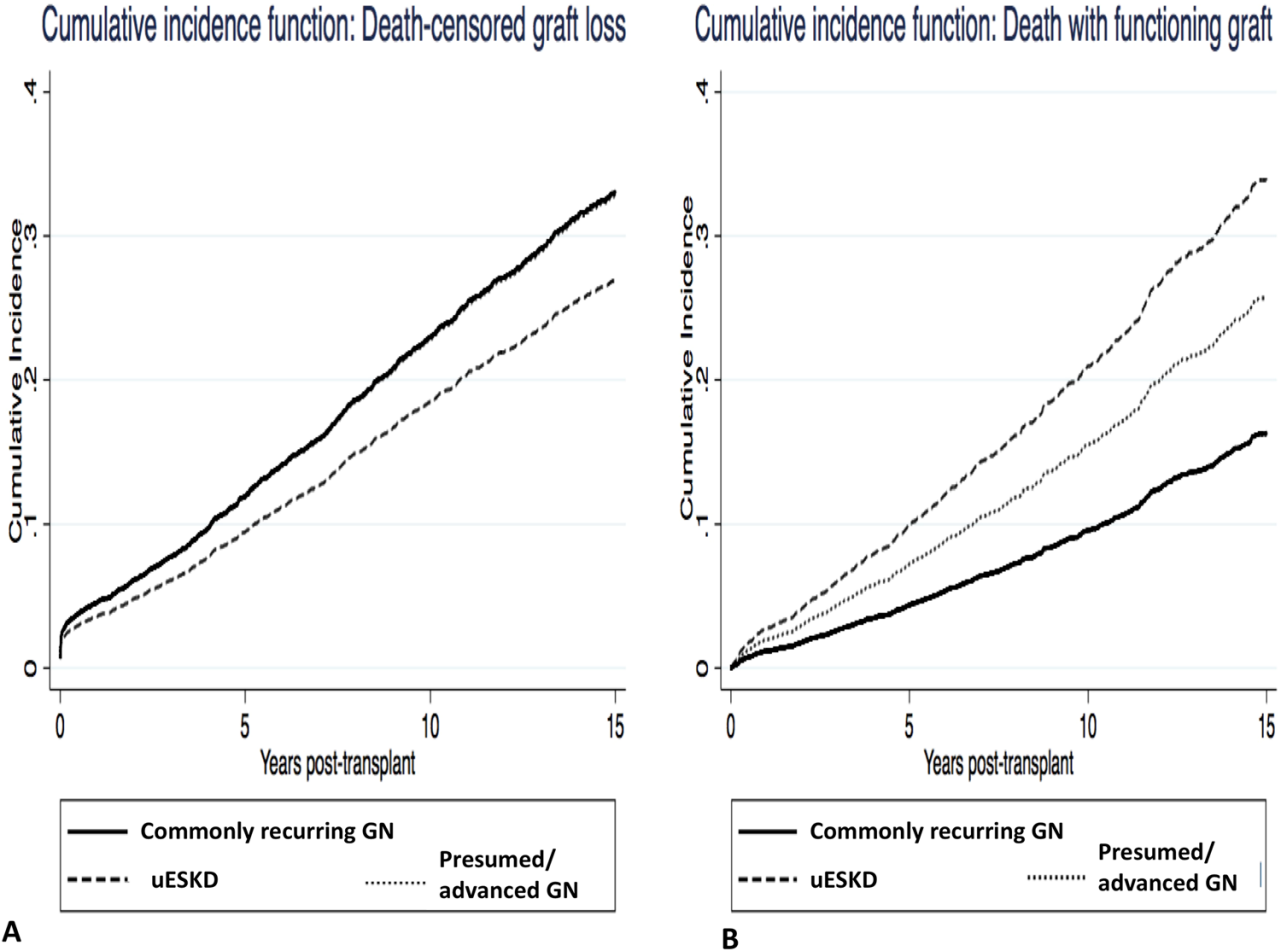
Cumulative incidence function curves of death censored graft failure (**A**) and death with a functioning graft (**B**) stratified by categories of end-stage kidney disease, adjusted for the competing risk of death with a functioning graft and death censored graft failure, respectively.

Compared to recipients with commonly-recurring GN, the adjusted subdistribution HR of DCGF for recipients with ESKD attributed to presumed/advanced GN and uESKD were 0.93 (0.78–1.12, p = 0.46) and 0.72 (0.54–0.96, p = 0.02), respectively (Table 2 and Fig. 1). Figure 3A shows the cumulative incidence of DCGF, stratified by ESKD groups, after adjusting for competing risk of DWFG.

### Associations between causes of ESKD and DWFG

Compared to recipients with commonly-recurring GN, the adjusted sub-distribution HR of DWFG for recipients with ESKD attributed to presumed/advanced GN and uESKD were 1.09 (0.87, 1.38, p = 0.44) and 1.58 (1.17, 2.15, p = 0.003), respectively (Table 2 and Fig. 1). Figure 3B shows the cumulative incidence of DWFG, stratified by ESKD groups, after adjusting for competing risk of DCGF.

### Associations between causes of ESKD and cause-specific DWFG

Compared to recipients with commonly-recurring GN, the incidence of DWFG attributed to cardiovascular disease (2.1%, vs. 4.8% and 4.8%, respectively; p < 0.001), infection (1.7% vs. 2.3% and 4.8%, respectively; p < 0.001) and cancer (3.8% vs. 5.6% and 5.9%, respectively; p = 0.005) were higher in recipients with presumed/advanced GN and uESKD. Compared to recipients with commonly-recurring GN, recipients with uESKD experienced a higher risk of cancer-related DWFG (adjusted sub-distribution HR 1.72, 1.07–2.74, p = 0.02), but not cardiovascular disease (adjusted sub-distribution HR 0.88, 0.40–1.84, p = 0.75) or infection-related DWFG (adjusted sub-distribution HR 1.57, 0.85–2.90, p = 0.15). There were no associations between presumed/advanced GN and any cause-specific DWFG.

### Associations between causes of ESKD and all-cause mortality

Patient survival at 5 and 10 years for recipients with commonly-recurring GN was 94% (93–95%) and 85% (83–86%), respectively. This compared with 88% (85–90%) and 75% (72–78%), respectively for recipients with presumed/advanced GN; and 86% (83–89%) and 75% (70–79%), respectively for recipients with uESKD (log-rank p < 0.001). In the unadjusted model, recipients with presumed/advanced GN or uESKD were more likely to die with unadjusted HR of 1.61 (1.41–1.84, p < 0.001) and 1.93 (1.61–2.32, p < 0.001), respectively compared to recipients with commonly-recurring GN. However, these associations were no longer significant in the adjusted model (Fig. 1).

## Discussion

For a significant proportion of people with ESKD, their cause of ESKD is not determined with certainty. ESKD is attributed to presumed/advanced GN in a proportion of these cases where clinical characteristics suggest GN however a specific diagnosis was not obtained because a biopsy was not performed or was non-diagnostic. Others are classified as ESKD of unknown cause. For both groups, GN remains a possible cause. Given that GN recurrence is one of the leading causes of graft loss after kidney transplantation, understanding the graft outcomes of such recipients would enable a more adequate discussion of risks and benefits, and specifically their risk of GN and GN-related graft failure, prior to kidney transplantation. We found that GN-related graft failure was an uncommon event among these two groups after kidney transplantation, affecting less than 1% after a median follow-up period of 8 years. Over the same period, 5% of those with a type of GN known to recur after transplantation experienced graft failure, which was attributed to recurrence. This finding remained robust after adjustment for potential confounders and thus such patients can be relatively reassured. Consistent with previous literature^7^, GN-related graft failure was second to chronic allograft nephropathy/interstitial fibrosis and tubular atrophy (CAN/IFTA) as the most common cause of DCGL among those with a GN type known to recur, whereas CAN/IFTA, acute rejection, vascular complications and other causes were all more common than GN-related graft failure for those with presumed/advanced GN or uESKD.

In considering transplantation, patients often have concerns beyond the risk that their original kidney disease may recur after kidney transplantation such as the risk of acute rejection, graft failure from any cause and death from cancer or other causes^14^. In this analysis, people with presumed/advanced GN or uESKD were no different to those with defined types of GN in terms of their risk of overall graft failure in adjusted models. Here, the higher rates of GN-related graft failure among those with recurring types of GN were negated by higher rates of graft failure attributed to a number of other causes among those with presumed/advanced GN and uESKD. Risks of all-cause mortality were modestly higher for those with presumed/advanced GN and uESKD as compared to known GN, however such differences were accounted for by factors including age and comorbid status and so became non-significant in the adjusted models.

Recurrence and/or de novo GN has been reported in up to 40% of kidney transplant recipients, with the cumulative incidence increasing with time post-transplant^3–6^. Those affected by recurrence incur a two-fold increase in risks of subsequent graft failure^7^, the majority of which is attributable to recurrence. The vast bulk of affected patients were those with biopsy-proven GN of a type known to recur. Risks for those with presumed/advanced GN are less well reported. In a prospective cohort study of 2606 kidney transplant recipients between 1990–2005, it was shown that only 6% of recipients who were classified as having presumed GN as cause of ESKD had biopsy-proven post-transplant GN, compared to 13% in those whose cause of ESKD was attributed to biopsy-proven GN pre-transplant. However, the authors did not specifically evaluate the risk of GN-related graft loss by GN subtypes^3^. After excluding those who died with a functioning graft, we found that 5% of recipients with presumed/advanced GN experienced GN-related graft failure, compared to 21% in those with commonly-recurring GN, with the majority (13/15, 87%) being classified as recurrent/de novo IgA nephropathy, FSGS or IMN. Despite the lower risk of GN-related graft failure in those with presumed/advanced GN compared to commonly-recurring GN, the risks of overall graft failure and DCGF were similar. We hypothesised that factors which precluded obtaining biopsy-confirmation of GN as a cause of ESKD may have also prevented a diagnosis of GN post-transplant in the presumed/advanced GN group. Such factors would likely yield an increase in graft failure attributed to CAN/IFTA, as this diagnosis is commonly made on the basis of clinical findings, such as proteinuria and/or declining eGFR, which may also be present in patients with GN^4^. However, the incidence of graft failure attributed to CAN/IFTA was equal in all groups (Table 3). One alternate hypothesis is that those with presumed/advanced GN, and also those with uESKD, may be more prone to non-adherence. We found a trend in this direction, as non-adherence was the attributed cause of graft failure for a numerically higher proportion of people in these groups as compared to known GN. Further work specifically examining adherence would be required to substantiate this point.

The reported incidence of uESKD (i.e. ESKD without an identifiable etiology or categorized as unknown chronic kidney disease [CKDu] in some countries) may be as high as 25%, often occurring in the absence of significant proteinuria or any identifiable risk factors^15–17^. With the incidence of uESKD being more common in specific geographical areas such as Central America, Sri Lanka, Egypt and India, it is likely that a combination of unspecified environmental, infectious and/or occupational exposures may be contributing to the development of this disease^10^. There is currently no uniformity and consensus with regards to the diagnostic criteria for uESKD, therefore reliable estimates of the risk of graft failure and/or death after kidney transplantation are unknown. In a large Canadian cohort study, 6% of kidney transplant recipients had uncertain cause of ESKD of whom 12% developed GN post-transplant^3^. Our study found that less than 10% of our study cohort had uESKD (or 4% of all incident primary kidney transplant recipients), of whom less than 1% experienced GN-related graft failure. In our study, recipients with uESKD were less likely to experience DCGF but had a higher risk of DWFG, possibly related to the residual confounding effects of older age and a greater proportion of recipients with comorbid conditions compared to recipients with commonly-recurring GN. However, it is possible that exposure to environmental or occupational toxins may be causal in some cases of uESKD and that these may also contribute to the excess risk of DWFG (including cancer-related DWFG) in this group. It is important to point out that uESKD as reported to the ANZDATA registry represents a constellation of varying diseases with dissimilar causative factors, and likely different to a diagnosis of CKDu, which is a distinct clinical entity in certain countries. A more in-depth review of the occupational and/or environmental exposure to toxins may help to establish the true etiology of uESKD, but this is beyond the scope of registry data.

Our study has several strengths and limitations. This is one of the largest cohort studies to examine the association between presumed/advanced GN and uESKD and long-term graft and patient outcomes in the era of modern immunosuppression. In capturing GN post-transplant, we have likely included recurrent and de novo disease, particularly in the presumed/advanced GN and uESKD groups. Selection bias is likely to exist because there may be systematic differences in the selection and listing of ESKD patients with different GN types as well as differences in the management of these recipients pre- and post-transplant. In addition, the cause of ESKD in the registry is assigned to the dominant cause, in the opinion of the nephrologist, which may not always be biopsy proven. Even though multiple confounding factors were adjusted for, it is likely that unmeasured residual confounders such as the severity of comorbidities, intensity of immunosuppression, and treatment of GN prior to transplants, which are not collected by ANZDATA registry may have modified the association between ESKD groups and outcomes. Because histological confirmation is not a requirement of the ANZDATA registry in the reporting of ESKD diagnosis or causes of graft loss, incorrect classification of causes of graft loss could potentially occur. Nevertheless, we have previously shown that a high proportion of cases of allograft failure, which are attributed to GN in ANZDATA registry, are indeed biopsy proven^7,8^. The relatively small number of GN-related graft losses and medium-term follow-up period may potentially have led to erroneous inference and therefore we are unable to generate reliable estimates with certainty.

Transplant candidates with presumed/advanced GN or ESKD of unknown cause are at relatively low-risk of incurring graft failure from post-transplant GN, as opposed to those with biopsy-proven GN of a type known to recur. As overall patient and graft survivals were similar between those with GN, presumed/advanced GN and uESKD, we propose that the cause of ESKD should not in general be used to distinguish between the three groups in terms of their access to transplantation.

## Materials and Methods

### Study population

Primary live and deceased donor kidney-only transplant recipients with ESKD secondary to GN or uncertain diagnosis in Australia and New Zealand between 1990–2012 were included. Of 12,859 patients with ESKD who have received primary kidney-only transplants, recipients with ESKD secondary to non-commonly-recurring GN (n = 752) and non-GN causes of ESKD other than uESKD (n = 6803) were excluded. The most common GN type in the non-commonly-recurring group is familial GN (n = 229 [30.5%]), followed by extra/intra-capillary GN (n = 135 [18.0%]), IgA-negative mesangial proliferative GN (n = 107 [14.2%]), Goodpasture’s syndrome (n = 102 [13.6%], secondary FSGS (n = 17 [2.3%]) and other GN with systemic causes (n = 162 [21.4%]). In the non-GN causes of ESKD (other than uESKD), 27% were attributed to cystic disease, 23% diabetic nephropathy, 18% reflux nephropathy, 8% vascular disease, 3% analgesic nephropathy and 21% other causes.

Of those with commonly-recurring GN, 46 (1.3%) were not reported to have biopsy-proven disease and were therefore excluded leaving a final cohort of 5258 recipients for analysis. The Strengthening the reporting of observational studies in epidemiology (STROBE) guideline for reporting is shown in Supplementary Fig. 1. The clinical and research activities being reported are consistent with the Principles of the Declaration of Istanbul as outlined in the ‘Declaration of Istanbul on Organ Trafficking and Transplant Tourism’. Approval of study by research ethics committee and informed consents were not required because only de-identified information were utilized for analysis. However, consents for inclusion in the ANZDATA registry were sought from all patients with ESKD in Australia and New Zealand.

### Exposure factor

Recipients were categorised according to the causes of ESKD: 1) Commonly-recurring GN (ESKD secondary to IgA nephropathy, MPGN, FSGS and IMN); 2) Presumed/advanced GN; and 3) uESKD (ANZDATA coded as “uncertain diagnosis”). Categories of ESKD collected by ANZDATA registry are listed in http://www.anzdata.org.au/forms/ANZDATA/ anzdata_A3_2013.pdf.

### Data collection

Baseline donor, recipient and transplant-related characteristics included donor age, type and gender; recipient age, gender, race, body mass index (BMI), waiting time pre-transplant, diabetes and coronary artery disease and peripheral vascular disease at time of transplantation; and transplant-related characteristics included peak percentage panel reactive antibody, induction therapy, number of human leukocyte antigen (HLA)-mismatches, total ischaemic time, transplant era and initial immunosuppressive agents.

### Clinical Outcomes

The primary outcome of this study was GN-related graft failure. Secondary outcomes included overall graft failure, death-censored graft failure (DCGF), death with a functioning graft (DWFG) and all-cause mortality. Data was censored as of 31^st^ December 2012. There were no missing outcome data for this study cohort.

### Statistical analyses

Data were expressed as number (proportion), mean ± standard deviation (SD) and median and interquartile range (IQR) where appropriate. Comparisons between categories of ESKD were made by chi-square test and analysis of variance (ANOVA) for categorical and continuous variables, respectively. The associations between categories of ESKD and outcomes were examined using adjusted Cox proportional hazards regression or competing risk analyses. The proportional hazards assumptions of all models were checked graphically by plotting the Schoenfeld residuals, and there were no evidence of departures from proportional hazards. The covariates with missing data are shown in the flow chart (Supplementary Fig. 1). The missing data for the covariates including total ischaemic time, peak PRA and HLA-mismatches were censored in the adjusted Cox regression and competing risk models (where appropriate); whereas the missing data for BMI, smoking status, prevalent coronary artery disease and peripheral vascular disease were considered as a separate category. Covariates with p-values of <0.10 in the unadjusted analyses were included in the multivariable-adjusted analyses, although transplant era, waiting time, ethnicity, donor and recipient age, diabetes status, smoking history and prevalent vascular disease (coronary artery disease and peripheral vascular disease) were included in all models because of their established biological relationships with graft and patient outcomes. Results were expressed as hazard ratio (HR) with 95% confidence interval (95%CI). Graft and patient survivals (with 95%CI) at 5 and 10 years post-transplant were determined using the Kaplan Meier method.

### Competing risk analysis

We conducted a competing risk regression for GN-related graft failure, DCGF, DWFG and cause-specific DWFG (cardiovascular disease [CVD], infection and cancer-related DWFG) taking into account the informative nature of censoring due to competing risk using the method of Fine and Gray^18^. For GN-related graft failure, allograft failures attributed to causes other than GN (including acute rejection-related allograft failure, CAN/IFTA-related allograft failure and other non-GN causes-related allograft failure) were considered as competing risks. The sub-distributional HRs and 95%CI were calculated to estimate the covariate effects on the cumulative incidence. Other covariates included in the competing risk models were identical to those included in the Cox regression models. Statistical evaluation was performed by SPSS V10 software program and STATA version 11. P-values of <0.05 were considered statistically significant.

### Data availability

The authors confirm that all data underlying the findings are fully available without restriction. The primary dataset for this manuscript was generated and made available to the authors by the Australian and New Zealand Dialysis and Transplant (ANZDATA) Registry, Adelaide, Australia. The ANZDATA Data Use Agreement between the ANZDATA Registry and the authors does not allow the authors to make the data publicly available. The authors confirm that all data underlying the findings can be obtained without restriction from the ANZDATA Registry. The interested researchers are advised to contact the ANZDATA Registry independently (email address requests@anzdata.org.au).

## Electronic supplementary material

Supplementary figure 1
