# Supplementary material for: Long-term outcomes of kidney transplant recipients with end-stage kidney disease attributed to presumed/advanced glomerulonephritis or unknown cause
Source: Sci Rep. 2018 Jun 13;8:9021. doi: 10.1038/s41598-018-27151-4 (PMC5998026; doi:10.1038/s41598-018-27151-4)

Long-term outcomes of kidney transplant recipients with end-stage kidney disease attributed to presumed/advanced glomerulonephritis or unknown cause

Wai H Lim [1,2,3*#], Germaine Wong [3,4,5,6], Stephen P McDonald [3,7], Aron Chakera [1,2], Grant Luxton [8], Nicole M Isbel [9], Helen L Pilmore [10], Tom Barbour [11], Peter Hughes [11], Steven J Chadban [3,12,13]

Supplementary figure 1. Flowchart for The Strengthening the reporting of observational studies in epidemiology (STROBE) guideline.

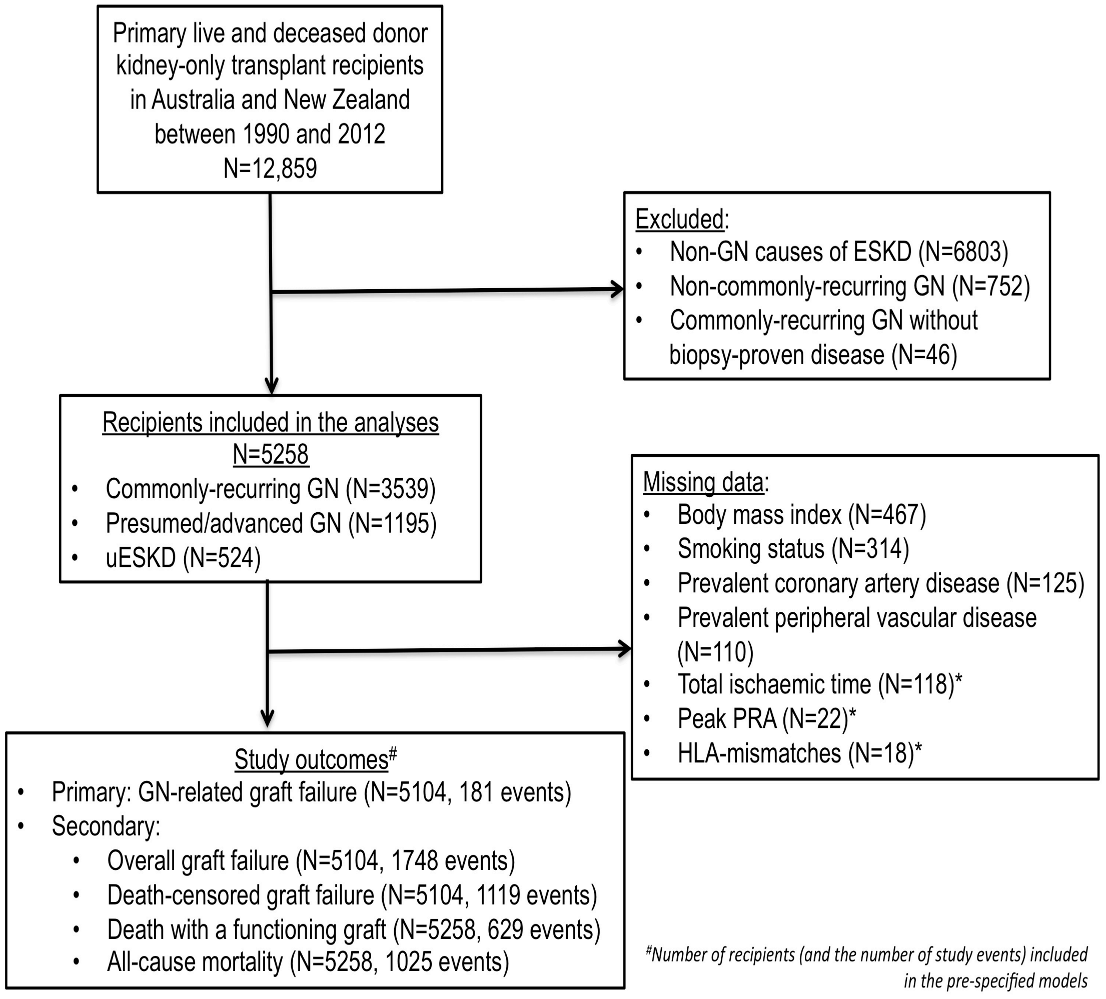

Supplement: Supplementary file 1 — Supplementary figure 1 [file 41598_2018_27151_MOESM1_ESM.pdf]
